# Detection of* Enterocytozoon bieneusi* in White Yaks in Gansu Province, China

**DOI:** 10.1155/2017/5790181

**Published:** 2017-06-06

**Authors:** Jian-Gang Ma, Nian-Zhang Zhang, Jun-Ling Hou, Yang Zou, Gui-Xue Hu, Xing-Quan Zhu, Dong-Hui Zhou

**Affiliations:** ^1^State Key Laboratory of Veterinary Etiological Biology, Key Laboratory of Veterinary Parasitology of Gansu Province, Lanzhou Veterinary Research Institute, Chinese Academy of Agricultural Sciences, Lanzhou, Gansu Province 730046, China; ^2^College of Animal Science and Technology, Jilin Agricultural University, Changchun, Jilin Province 130118, China; ^3^Jiangsu Co-Innovation Center for the Prevention and Control of Important Animal Infectious Diseases and Zoonoses, Yangzhou University College of Veterinary Medicine, Yangzhou, Jiangsu Province 225009, China

## Abstract

*Enterocytozoon bieneusi*, the most common zoonotic pathogen of microsporidiosis, has been found in various animals and humans, but no information is available concerning the prevalence and genotypes of* E. bieneusi *in white yaks* (Bos grunniens)*. In the present study, 353 faecal samples from white yaks in Tianzhu Tibetan Autonomous County, Gansu Province, Northwestern China, were collected and examined by PCR amplification of the internal transcribed spacer gene to estimate* E. bieneusi* prevalence and identify their genotypes. Of the 353 faecal samples, 4 (1.13%) were tested* E. bieneusi*-positive. Sequences analysis revealed that two known genotypes, namely, I (*n* = 1) and BEB4 (*n* = 2), and a novel genotype, namely, WCY1 (*n* = 1), were found in this study. Among them, genotype WCY1 was clustered into Group 1, and genotypes I and BEB4 belonged to Group 2. The present study firstly indicates the existence of* E. bieneusi* in yaks in Gansu Province, Northwestern China. This is also the first record of* E. bieneusi* in white yaks. Effective measures should be taken to control* E. bieneusi* infection in white yaks, other animals, and humans.

## 1. Introduction

Microsporidiosis is caused by Microsporidia, which have been recognized as opportunistic pathogens for humans and animals [1, 2]. More than 1500 microsporidian species in 190 genera have been found since 1985 [3]. Among them, 14 species in 8 genera have been identified in humans [4]. Of these,* Enterocytozoon bieneusi* is the most common causative agent of human infection [5], with the symptoms of chronic diarrhea and other enteric disease [6].

At present, over 200 distinct genotypes of* E. bieneusi* have been identified based on ribosomal internal transcriber spacer (ITS) region of the rRNA gene [7]. These genotypes could be divided into several genetically isolated clusters, including a large cluster named as “the zoonotic genotypes” (Group 1) and some other groups, the so-called “host adapted groups” (Groups 2 to 9) [7]. It is surprising that some of the genotypes (I, J, and BEB4) in Group 2 have also been found in humans in recent years [8–10], so investigations of* E. bieneusi* prevalence in different hosts and assessing their zoonotic potential are important.

In China, the prevalence and genotypes of* E. bieneusi *have been reported in humans, some animals, and wastewater [11–13]. The yak is a unique bovine species and is a valuable, semiwild animal, living at high altitudes. The majority of yaks are distributed in China. As a unique yak breed, the white yak lives only in the Tianzhu Tibetan Autonomous County (TTAC), Gansu Province, Northwest China. However, no information regarding* E. bieneusi* prevalence in white yaks is available. Therefore, the objective of the present study was to estimate prevalence and genotypes of* E. bieneusi* in white yaks in China.

## 2. Materials and Methods

### 2.1. Specimen Collection

Between June 2013 and October 2016, 353 fresh faecal specimens of white yaks in two farms in Tianzhu Tibetan Autonomous County (TTAC), Gansu Province, Northwest China, were collected. One faecal sample was collected from each examined white yak, and the basic information was recorded in Table 1. All samples were collected using a sterile disposable glove and then were stored in box with ice immediately and sent to the laboratory.

### 2.2. PCR Amplification

The DNA samples of feces were extracted with E.Z.N.A.® Stool DNA Kit (OMEGA, USA) according to the manufacturer's instructions. Then the DNA samples were stored at −20°C until used. PCR amplification was performed using the nested primers specific for the ITS region of* E. bieneusi* reported in the previous study [12]. The reaction mixture (25 *μ*l) consisted of 1x Ex* Taq* buffer (Mg^2+^ free), 200 *μ*M dNTPs, 2 mM MgCl_2_, 0.4 *μ*M of each primer, 0.625 U Ex* Taq* DNA polymerase (TAKARA, Japan), and 2 *μ*l of DNA sample. After preheating at 94°C for 5 min, the reaction was conducted for 35 cycles at 94°C for 45 s, 55°C for 45 s, and 72°C for 1 min, with a final extension at 72°C for 10 min. All the PCR products were electrophoresed in 2% agarose gels containing ethidium bromide and then visualized under UV light.

### 2.3. Sequencing and Phylogenetic Analyses

**Figure 1:**
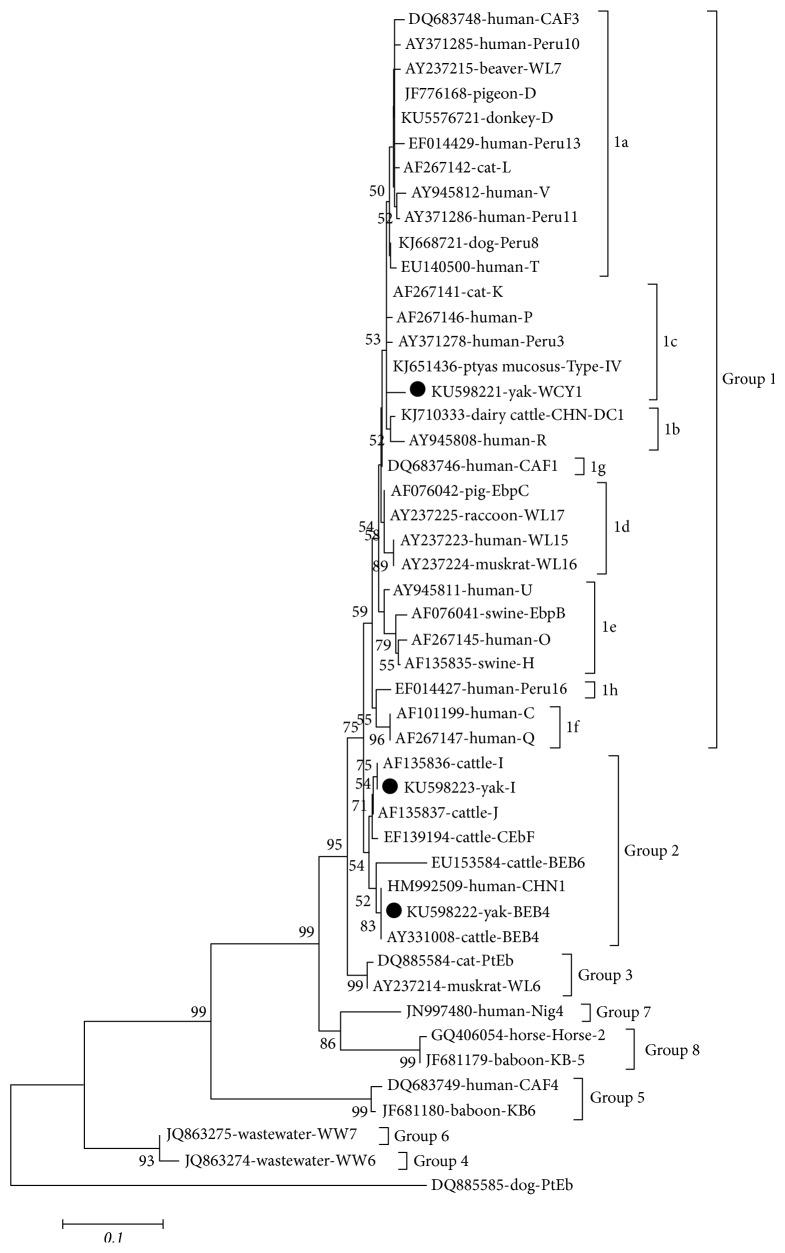
Phylogenetic analyses of the* E. bieneusi* genotypes based on sequences of the internal transcribed spacer (ITS) using the neighbor-joining (NJ). Bootstrap values > 50% are shown. The genotypes identified in this study are indicated by ●.

The positive secondary PCR were sequenced by Genscript Company (Nanjing, China). The sequences were confirmed by the program Clustal X 2.0 and aligned with reference sequences of* E. bieneusi* available in GenBank to determine the genotypes. Neighbor-joining (NJ) method was used to reconstruct the phylogenetic tree, and bootstrap analysis was performed using 1,000 replicates (Figure 1). The novel genotype(s) of* E. bieneusi *was named based on the established nomenclature system.

### 2.4. Nucleotide Sequence Accession Numbers

The representative nucleotide sequences of this study have been deposited in the GenBank database under accession number KU598221-598223.

## 3. Results

Of the 353 yak faecal samples, 4 (1.13%) were* E. bieneusi*-positive tested by nested PCR. The prevalence of* E. bieneusi* in farm 1 was 5.26% (4/76), with 0% (0/277) in farm 2. Moreover, the prevalence in summer and autumn was 0% (0/123) and 1.73% (4/230), respectively (Table 1).

A total of 3 genotypes, including 2 known genotypes, I (*n* = 1) and BEB4 (*n* = 2), and one novel genotype, WCY1 (*n* = 1), were identified in the present study (Table 1). Phylogenetic analysis revealed that genotype WCY1 was subclustered into Group 1c, whereas genotypes I and BEB4 were classified into Group 2.

## 4. Discussion

The overall prevalence of* E. bieneusi* in white yaks in China was 1.13%, which was lower than that in black yaks in Qinghai (7%, 23/327) [6], dairy cattle in Heilongjiang (30.1%, 40/133) [14], dairy cattle in Harbin, Daqing, and Qiqihar (6%, 32/537) [4], dairy cattle in Henan and Ningxia (24.3%, 214/879) [7], and cattle in Shaanxi (19.68%, 73 of 371) [15] but slightly higher than in domestic rabbits in the northeast China (0.94%, 4/426) [12]. It is also lower than that reported in dairy cattle in Argentina (14.3%, 10/70) [16], cattle in USA (34.8%, 285/819) [17], and cattle in Brazil (17.5%, 79/452) [18]. These differences may be influenced by many factors, such as the sample size, animal species, and the region of sample collected, so that it is difficult to explain the real reasons among different studies.

In this study, three ITS genotypes (two known genotypes and a novel genotype) were identified in white yaks in China, with BEB4 being the predominant genotype, which is consistent with that reported in black yaks in Qinghai [6]. The present study suggests that the three genotypes (I, BEB4, and WCY1), identified in this study, together with the genotypes J, CHN11, and CHN12, previously found in black yaks in Qinghai, are endemic in yaks in China. Both the known genotypes BEB4 and I identified in this study were also previously reported in nonhuman primates (genotypes BEB4 and I) and cats (genotype I) in China [19]. Sequence analysis indicated that the novel* E. bieneusi *genotype (WCY1) is closely related to genotype GD-9 (KF305586) from cynomolgus monkey, with three nucleotide differences, and it has four nucleotide differences relative to the sequence of genotype PSD-16 (KJ668720) from dog. The remaining genotypes (KU598222 and KU598223) have identical sequences to the reference genotypes BEB4 (AY331008) and I (KT984486) available in GenBank. Of the three genotypes, genotype WCY1 belonged to Group 1, which implied that the white yaks may be a potential source of human infection. Although the remaining genotypes I and BEB4 were classified into Group 2, we must be cautious because the cattle-specific genotypes (I, BEB4, and J) have been found in humans [8–10].

In conclusion, the present study demonstrates the occurrence of* E. bieneusi* in white yaks for the first time. Moreover, the present study also indicates that the genotypes I, WCY1, and BEB4, found in this study, together with the genotypes J, CHN11, and CHN12, previously identified in black yaks in Qinghai, are endemically prevalent in yaks in China.

## Figures and Tables

**Table 1:** Prevalence and genotypes of *E. bieneusi* in white yaks in Gansu Province, Northwestern China.

Farm	Season	Number tested	Number positive (%)	Genotype (*n*)
Farm 1	Autumn	76	4 (5.26)	WCY1 (*n* = 1), BEB4 (*n* = 2), I (*n* = 1)
Farm 2	Summer	123	0 (0)	—
	Autumn	154	0 (0)	—
Total		353	4 (1.13)	WCY1 (*n* = 1), BEB4 (*n* = 2), I (*n* = 1)
